# Comparison of Fetal Crown-Rump Length Measurements between Thawed and Fresh Embryo Transfer

**DOI:** 10.3390/jcm13092575

**Published:** 2024-04-27

**Authors:** Kyriaki Mitta, Ioannis Tsakiridis, Evaggelia Giougi, Apostolos Mamopoulos, Ioannis Kalogiannidis, Themistoklis Dagklis, Apostolos Athanasiadis

**Affiliations:** Third Department of Obstetrics and Gynaecology, School of Medicine, Faculty of Health Sciences, Aristotle University of Thessaloniki, 54642 Thessaloniki, Greece; kmittb@auth.gr (K.M.); egiougi@gmail.com (E.G.); amamop@auth.gr (A.M.); ikalogia@auth.gr (I.K.); dagklis@auth.gr (T.D.); apathana@auth.gr (A.A.)

**Keywords:** ART, thawed, fresh, embryo, CRL, measurement

## Abstract

**Background and Objectives:** Neonates born from thawed embryo transfers tend to have a significantly higher birthweight compared to those from fresh embryo transfers. The aim of this study was to compare the crown-rump length (CRL) between thawed and fresh embryos to investigate the potential causes of different growth patterns between them. **Materials and Methods:** This was a retrospective study (July 2010–December 2023) conducted at the Third Department of Obstetrics and Gynecology, School of Medicine, Faculty of Health Sciences, Aristotle University of Thessaloniki, Greece. In total, 3082 assisted reproductive technology (ART) pregnancies (4044 embryos) underwent a routine scan at 11^+0^–13^+6^ gestational weeks and were included in the study. Maternal age, the type of embryo transfer (thawed vs. fresh, donor vs. their own oocytes), CRL, twin and singleton gestations were analyzed. **Results:** The mean maternal age in thawed was significantly higher than in fresh embryos (39.8 vs. 35.8 years, *p*-value < 0.001). The mean CRL z-score was significantly higher in thawed compared to fresh embryo transfers (0.309 vs. 0.199, *p*-value < 0.001). A subgroup analysis on singleton gestations showed that the mean CRL z-score was higher in thawed blastocysts compared to fresh (0.327 vs. 0.215, *p*-value < 0.001). Accordingly, an analysis on twins revealed that the mean CRL z-score was higher in thawed blastocysts (0.285 vs. 0.184, *p*-value: 0.015) and in oocytes’ recipients compared to own oocytes’ cases (0.431 vs. 0.191, *p*-value: 0.002). **Conclusions:** The difference in CRL measurements between thawed and fresh embryos may be a first indication of the subsequent difference in sonographically estimated fetal weight and birthweight. This finding highlights the need for additional research into the underlying causes, including maternal factors and the culture media used.

## 1. Introduction

The use of assisted reproductive technology (ART) is constantly rising, and thus associated fetal, maternal and neonatal complications have been investigated over the past few years. Recent findings have indicated an increased risk of low birthweight in infants born after fresh cycle in vitro fertilization (IVF)/intracytoplasmic sperm injection (ICSI) treatments [1]. Studies comparing the outcomes of pregnancies resulting from thawed and fresh blastocysts following IVF/ICSI have shown that infants born from thawed blastocysts have significantly higher birthweight than those born from fresh embryo transfers [2].

Regarding the health outcomes of infants conceived via ART, the current literature suggests that IVF/ICSI is associated with an increased likelihood of small-for-gestational age (SGA) neonates, as well as higher incidences of low-birthweight and preterm births [1,3]. Notably, thawed embryos are associated with large-for-gestational age (LGA) neonates and a higher risk of gestational hypertension and preeclampsia [4]. Moreover, the risks of antepartum hemorrhage, SGA, preterm birth, low birthweight and perinatal mortality are all significantly lower in pregnancies from thawed embryo transfers [3]. Thawed embryo transfer at the blastocyst stage is suggested to be the most suitable method in terms of optimal fetal growth [5].

In most settings, a first-trimester scan is offered at 11^+0^–13^+6^ gestational weeks for dating, screening for preeclampsia and chromosomal abnormalities and the early detection of severe fetal defects [6,7]. The aim of this study was to compare the crown-rump length (CRL) between thawed and fresh embryos at a routine first-trimester scan. Taking into consideration the different fetal growth patterns and birthweights between the two types of embryos, an investigation of the potential differences in CRL measurements could clarify the reasons for these discrepancies and signify when these changes occur.

## 2. Materials and Methods

This was a retrospective study (July 2010–December 2023) conducted at the Third Department of Obstetrics and Gynecology, School of Medicine, Faculty of Health Sciences, Aristotle University of Thessaloniki, Greece. Maternal age, type of embryo transfer (thawed vs. fresh, donor vs. own oocytes), CRL and type of pregnancy (twin vs. singleton gestations) were recorded, as well. Inclusion criteria were women who underwent IVF using either fresh or frozen embryo blastocysts, regardless of the ovarian stimulation protocol, the number of embryos transferred or hormone levels involved. The pregnancies were confirmed to be between 11^+0^ and 13^+6^ weeks of gestation and all the participants had undergone a first-trimester scan performed by an expert sonographer. Any cases with anatomical or chromosomal abnormalities during the first-trimester scan, as well as all monochorionic twin gestations, were excluded from the study, as these may affect fetal growth. Those with missing data (i.e., type of embryo transfer) were also excluded.

All women had given their informed consent that their anonymized data could be used for future research. As per standard policy for audit or observational database studies not involving any intervention or modification of the management of the participants, no institutional review was required or obtained [8]. Of note, no incentives were provided to the participants. All first-trimester scans were performed by experienced maternal–fetal medicine specialists, according to the International Society of Ultrasound in Obstetrics and Gynecology guideline for first-trimester scans [9].

Qualitative variables were described as n (%), and quantitative data as mean (SD). An independent Student’s *t*-test was employed to compare the means of quantitative variables (maternal age, CRL z-score). CRL was expressed as z-scores of a reference population of 11–13^+6^ weeks (45–84 mm) in fresh and thawed/thawed blastocysts after ART conceptions [10]. CRL z-scores were calculated using the formula Z-score = (XGA − MGA)/SDGA, where XGA was the actual fetal CRL measurement at a given gestation and MGA and SDGA were the expected mean and SD according to the international standards for early fetal size and pregnancy dating based on an ultrasound measurement of crown-rump length in the first trimester of pregnancy, obtained from the general population [11]. Further, subgroup analyses were conducted according to the type of oocyte used (own vs. donor) and the type of pregnancy (singletons vs. twins). A chi-squared test (x^2^) was also employed to investigate any association between the types of gestations (twin vs. singleton) and the types of embryos transferred (thawed vs. fresh embryo transfer). It was also employed to examine any association between the type of gestation and the type of embryo (embryos from donor oocytes vs. their own ones). The level of statistical significance was set at *p* = 0.05. The statistical package IBM SPSS Statistics 29.0 was used.

## 3. Results

In total, 3082 ART pregnancies, including 4044 embryos, underwent a first-trimester scan during the study period. Of these embryos, 1255 (40.7%) originated from thawed transfers, whereas 1827 (59.3%) originated from fresh ones. The thawed embryos included 892 (71%) singleton pregnancies and 363 pairs of twins (726 embryos) (29%). The fresh embryos included 1228 (67.2%) singleton pregnancies and 599 pairs of twins (1198 embryos) (32.8%) (Table 1a,b). Of note, 1725 (56%) pregnancies were from donor and 1357 (44%) from own oocytes. The donor embryo transfers included 600 (35%) twin and 1125 (65%) singleton gestations. The own oocyte embryo transfers included 362 (27%) twin and 995 (73%) singleton gestations.

**Figure 1:**
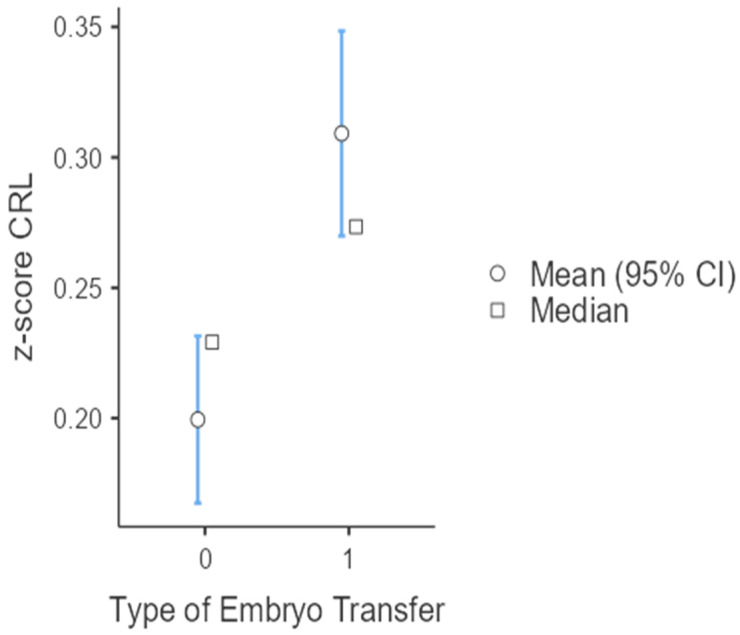
CRL z-score according to type of embryo transfer (ET) in the whole sample (0: fresh embryo transfer; 1: thawed embryo transfer).

The mean age of women who underwent thawed embryo transfer was higher compared to those following fresh embryo transfer (39.8 years vs. 35.8 years, *p* < 0.001). The incidence of twin gestations was significantly higher in fresh compared to thawed embryo transfer (OR:1.20, 95% CI: 1.06–1.36, *p*-value= 0.005). The CRL z-score was higher in thawed compared to fresh embryos in the total sample population (0.309 vs. 0.199, *p* < 0.001) (Table 1a,b, Figure 1).

**Figure 2:**
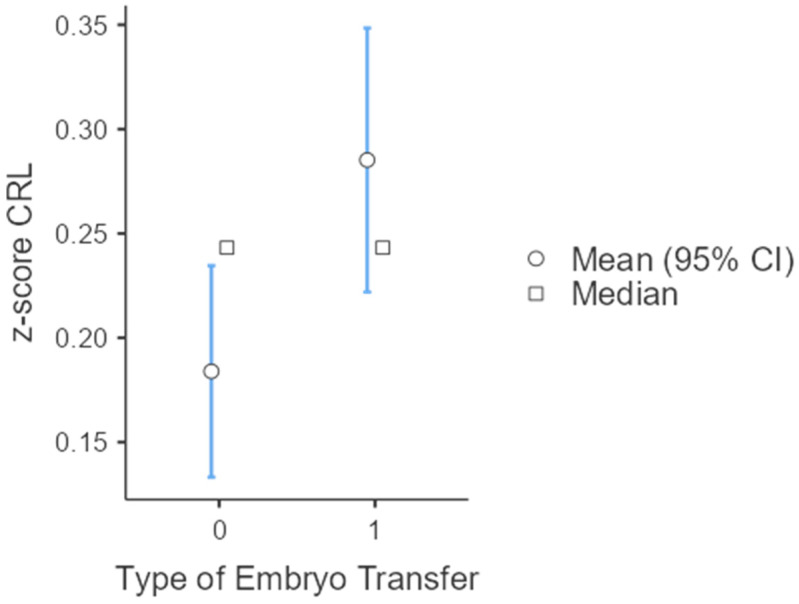
CRL z-score, according to type of embryo transfer (ET) in twin gestations (0: own oocytes; 1: donor oocytes).

**Figure 3:**
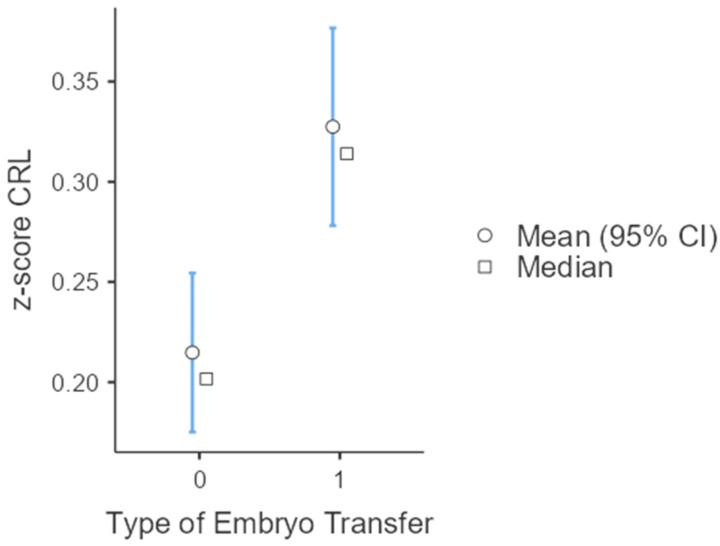
CRL z-scores according to type of embryo transfer in singleton gestations (0: fresh embryo transfer; 1: thawed embryo transfer).

A subgroup analysis on twin gestations found that the mean CRL z-score was significantly higher in both thawed cycles (0.285 vs. 0.184, *p*-value: 0.015) and in donor oocytes (0.431 vs. 0.191, *p*-value: 0.002) (Table 2, Figure 2). An analysis on singletons also showed that the mean CRL z-score was significantly higher in thawed compared to fresh cycles (0.327 vs. 0.215, *p*-value < 0.001) (Table 3, Figure 3).

## 4. Discussion

The main findings of this study were that (i) the mean maternal age in thawed embryo transfers was significantly higher than in fresh embryo transfers in the total sample, in singleton and twin gestations; (ii) the mean CRL z-score at 11^+0^–13^+6^ weeks was significantly higher in thawed embryos compared to fresh embryos in both singleton and twin gestations; (iii) the mean maternal age of donor oocytes cases was significantly higher than those who used their own oocytes in both singleton and twin gestations; (iv) twin gestations were significantly higher in fresh blastocysts; and (v) the mean CRL z-score was significantly higher in twin gestations, arising from donor oocytes.

The conventional IVF/ICSI procedure consists of a fresh embryo transfer, followed by thawed embryo transfers in subsequent cycles in the case of failure at the first attempt. However, an alternative approach consists of the “freeze-all” strategy, in which case all embryos are thawed and transferred in subsequent cycles. A meta-analysis, comparing the two approaches, showed that the cumulative live birth rate and ongoing pregnancy rate are the same in the two methods [12]. However, a decreased risk of ovarian hyperstimulation syndrome (OHSS) seems to be associated with the “freeze-all” strategy. The same meta-analysis showed that in large-for-gestational age neonates, birthweight and incidence of hypertensive disorders may be increased in the “freeze-all” strategy [12]. New evidence suggests that hypertensive disorders are increased only in frozen cycles after hormone-replacement therapy, due to the absence of the corpus luteum and the absence of relaxin; the latter is associated with systematic vasodilation and increased arterial compliance [13]. However, the available data in the literature suggest that the maternal and perinatal outcomes are, in general, improved in thawed compared to fresh embryo transfer [14]. Therefore, there is an increased tendency towards thawed embryo transfer, nowadays.

Higher maternal age is related to more obstetric and maternal complications; therefore, it is reasonable to proceed with thawed embryo transfer [14]. The maternal age is higher in thawed compared to fresh embryo transfers, according to a retrospective cohort study [15]. The same finding was supported by other published data [16], which were in agreement with the results of our study. Pregnancies resulting from thawed embryos have become increasingly prevalent in Europe, resulting in about half of the ART cycles in numerous countries [17], especially in cases involving oocyte donation [18]. The use of thawed embryo transfer offers several clinical advantages, including the avoidance of endometrial asynchrony [19,20].

A retrospective analysis of 5406 embryos, revealed that the birthweight of neonates from thawed embryos was higher than in those arising from fresh embryos, but the potential mechanisms of this difference were not elucidated [21]. Therefore, investigating any difference in CRL measurement between thawed and fresh embryos could partially clarify these mechanisms. According to a secondary analysis of a prospective cohort study, the average CRL z-score between 6 and 14 weeks was notably higher in thawed compared to fresh transfers; the likelihood of having a CRL below the fifth percentile was 68% for fresh versus 40% for thawed embryos at 6 weeks, and 2% versus 1% at 14 weeks, respectively [22]. Similarly, in our study, CRL measurement during the first trimester anomaly scan was significantly increased in thawed compared to fresh embryos. This difference remained significant in subgroup analyses in singletons and twins. Several factors could contribute to the observed differences in CRL growth, such as changes in the condition of the endometrium [23,24], epigenetic modifications in the trophoblast [25], variations in ovarian hormone levels during the peri-implantation phase [26], the quality of the embryo, differences in blood flow to the uterus and disparities in cardiovascular health among the groups studied [27]. Another factor that could explain this difference in CRL measurements between thawed vs. fresh embryos could be the freezing and thawing process itself; embryos that survive this process are often considered to be more robust, potentially contributing to the observed differences in early growth as measured by CRL. Notably, the high levels of hormones after ovarian stimulation in fresh cycles compared to frozen cycles may contribute to these discrepancies in CRL measurement, as well. The association between fetal growth and the type of embryo transfer in the first trimester of pregnancy indicates that there might be a link between endometrial environment, hormonal milieu and fetal growth, as suggested by published data [28]. Nonetheless, a definitive causal relationship explaining the growth patterns and abnormalities in fetuses from thawed or fresh embryos has not been established, suggesting the causes are likely varied and complex. Of note, the CRL measurement has been suggested in the literature as a potential prognostic factor for pregnancy outcome; pregnancies following spontaneous conception with higher CRL measurements had a significantly lower risk of stillbirth [29].

According to the results of our study, the maternal age of oocyte recipients was significantly increased compared to those who used their own, which is reasonably explained; in 2014, up to 12% of all IVF cycles in the U.S. were performed using donor oocytes and a high pregnancy rate from women in their 50s was observed (>35%) [30]. Oocyte donation currently represents the main option for aging-related infertility [30].

Twin gestations are still a relatively common occurrence in ART [31]. The results of our study indicated a higher prevalence of twin gestations in fresh compared to thawed embryo transfer; this could be explained by the number of embryos transferred in each case. Furthermore, a comparative study on the cost-effectiveness of single embryo transfer followed by an additional thawed embryo transfer, compared to double embryo transfer, showed that in women above 32 years of age, double embryo transfer was more effective in fresh cycles [32], explaining the higher prevalence of twins in fresh embryo transfer. Increasing the success rate in the area of ART is crucial, taking into consideration the high cost of the procedure [33]. A meta-analysis of individual patient data analysis from randomized trials showed that elective single embryo transfer is related to a higher chance of delivering a full-term, healthy neonate compared to double embryo transfer, yet it is also associated with a lower pregnancy rate in fresh cycles [13]. Even though this difference is completely overcome by an additional thawed embryo transfer, there is still a tendency towards maximizing the chances of live birth at the first attempt and not cumulatively [13]. Elective single embryo transfer is the only effective way of minimizing the risk of multiple gestations and can also be applied in women 36–39 years old [34]. Preimplantation genetic testing for aneuploidies (PGT) could also enhance the strategy of elective single embryo transfer, without compromising the results of ART. Although evidence suggests that PGT improves live birth rates in women above 35, the Greek legislation does not allow its implementation in women before 40 years of age, without any other indication, thus discouraging elective single embryo transfer [35].

Notably, the mean CRL z-score was significantly higher in cases of twin gestations arising from donor oocytes, which was not observed in singletons. The current literature suggests that CRL is higher in heterologous embryos, derived from donor oocytes, compared to homologous embryos, derived from the parent’s own gametes [36]. This could be attributed to the fact that donor gametes might be selected based on certain desirable traits, and overall vitality, which could have an impact on early embryonic growth, as indicated by CRL measurement. Furthermore, the age of the oocytes might have an impact, as well, since donor oocytes arise typically from young women. Therefore, oocytes from young donors may have a higher quality and better growth pattern due to the lack of genetic abnormalities. Sperm quality might affect the embryo’s early growth and development, as well. Sperm donors are selected through thorough screening processes. Regarding embryological techniques, differences in culture conditions between homologous and heterologous embryos could also play a role. Techniques might vary in donor programs within IVF clinics. Selection and embryo transfer practices might influence early growth, as well. The best-quality embryos are chosen more rigorously in donor cycles, compared to homologous cycles, due to the associated costs and implications. The hormonal environment, endometrial receptivity and hormonal preparation for IVF in donor–recipient cycles may be different, contributing to a more favorable environment for optimal early embryo growth. Further research is needed to determine the reasons why these differences in early growth exist. Moreover, in twin gestations, there might be interactions between embryos that influence their developmental capacity; these interactions might enhance growth patterns, such as CRL, especially in a highly optimized environment of donor embryo transfer. Maternal hormonal and immune system adaptations in response to embryo implantation could differ significantly between one and two embryos and might be influenced further by the genetic unfamiliarity of donor embryos. In twin pregnancies resulting from IVF, particularly with heterologous embryos, there may be a selection bias towards implanting the best-quality embryos, given the increased complexity of managing twin pregnancies. This selection could be even more pronounced with donor embryos, where there might be a higher availability of high-quality embryos due to factors like donor age and health. Differences in the genetics between heterologous and homologous embryos could be more pronounced in twins. Each embryo expresses different traits strongly influenced by the donor’s genetics, which might not be as evident in singleton pregnancies where intrauterine competition and resources are not factors.

The major strength of our study is its sample, which provides significant statistical power regarding the results. To date, this is the only study providing data on the differences between CRL in thawed and fresh embryos during the nuchal translucency scan. The main limitation of this study is its retrospective nature and other maternal co-factors (confounders) that could be a potential explanation for the findings, such as comorbidities, i.e., cardiovascular disease, including hypertension, body mass index and smoking. Of note, the risk of bias was eliminated because of the electronic recording of the data. Furthermore, with regard to the process, the patients underwent varying ovarian stimulation protocols and embryo transfer policies at different IVF units. Most of these patients did not provide comprehensive written medical information about the procedures, leading to a lack of accurate data on hormone levels, the number of oocytes retrieved and the type of ovarian stimulation used. As a result, this information was not included in our study.

## 5. Conclusions

A higher CRL measurement was found in cases of thawed compared to fresh embryos in both singleton and twin gestations. Knowing the precise day of conception in pregnancies achieved via ART, due to the specific timing of oocyte retrieval and subsequent embryo transfer, provides a strong reason to avoid dating these pregnancies using first-trimester sonographic measurements of CRL. A significant difference in the measurement of CRL between thawed and fresh embryos renders it necessary to further investigate the potential reasons for this, in terms of maternal factors and culture material. More longitudinal studies are encouraged to evaluate the impact of thawed embryos on the incidence of LGA fetuses.

## Figures and Tables

**Table 1:** (**a**) Baseline characteristics according to type of embryo transfer (thawed vs. fresh) for the whole sample. (**b**) Baseline characteristics according to type of embryo transfer (thawed vs. fresh) for the whole sample (chi-squared test).

(**a**)					
**Characteristics**	**Thawed Cycles**	**Fresh Cycles**	***p*-Value**		
Number of embryos	1618 (40%)	2426 (60%)	N/A		
Maternal age (mean/SD)	39.8 (5.7)	35.8 (4.3)	**<0.001**		
CRL z-score (mean/SD)	0.309 (0.806)	0.199 (0.805)	**<0.001**		
(**b**)					
**Characteristics**	**Thawed Cycles**	**Fresh Cycles**	***p*-Value**	**OR**	**95% CI**
Number of singleton gestations	892 (71%)	1228 (67.2%)			
Number of twin gestations	363 (29%)	599 (32.8%)	**0.005**	1.20	1.06–1.36

CRL: crown-rump length; N/A: not applicable. OR: odds ratio; 95% CI: 95% confidence interval.

**Table 2:** Subgroup analysis on twin gestations.

**Characteristics**	**Thawed Cycles**	**Fresh Cycles**	***p*-Value**
Maternal age (mean/SD)	40.1 (6.1)	35.1 (4.3)	**<0.001**
CRL z-score (mean/SD)	0.285 (0.869)	0.184 (0.893)	**0.015**
**Characteristics**	**Own Oocytes**	**Donor Oocytes**	***p*-Value**
Maternal age (mean/SD)	37.5 (5.1)	43.5 (4.7)	**<0.001**
CRL z-score (mean/SD)	0.191 (1.05)	0.431 (0.813)	**0.002**

CRL: crown-rump length.

**Table 3:** Subgroup analysis on singleton gestations.

**Characteristics**	**Thawed Cycles**	**Fresh Cycles**	***p*-Value**
Maternal age (mean/SD)	39.6 (5.3)	36.4 (4.3)	**<0.001**
CRL z-score (mean/SD)	0.327 (0.751)	0.215 (0.709)	**<0.001**
**Characteristics**	**Own Oocytes**	**Donor Oocytes**	***p*-Value**
Maternal age (mean/SD)	37.6 (4.5)	43.7 (4.1)	**<0.001**
CRL z-score (mean/SD)	0.455 (1.17)	0.314 (0.919)	0.412

CRL: crown-rump length.

## Data Availability

Data are available upon request.
